# Adaptive Optics Flood Illumination Ophthalmoscopy in Nonhuman Primates: Findings in Normal and Short-term Induced Detached Retinae

**DOI:** 10.1016/j.xops.2023.100316

**Published:** 2023-04-20

**Authors:** Alexandre Dentel, Elena Brazhnikova, Nathaniel Norberg, Céline Jaillard, Kate Grieve, Michel Paques, José A. Sahel, Stéphane Bertin, Valérie Forster, Serge Picaud

**Affiliations:** 1 Institut de la Vision, INSERM, CNRS, Sorbonne Université, Paris, France; 2 CHNO des Quinze-Vingts, INSERM-DGOS CIC 1423, Paris, France; 3 Department of Ophthalmology, University of Pittsburgh School of Medicine and Medical Center, Pittsburgh, Pennsylvania

**Keywords:** Adaptive optics, Nonhuman primates, Retinal detachment, rtx1

## Abstract

**Objective:**

To describe adaptive optics flood illumination ophthalmoscopy (AO-FIO) of the photoreceptor layer in normal nonhuman primates (NHPs) and in the case of a short-term induced retinal detachment (RD).

**Design:**

Longitudinal fundamental research study.

**Subjects:**

Four NHPs were used to image normal retinae with AO-FIO (in comparison with 4 healthy humans); 2 NHPs were used to assess the effects of RD.

**Intervention:**

The photoreceptor layer (cone mosaic metrics, including cone density, cone spacing, and cone regularity) was followed with AO-FIO imaging (rtx1, Imagine Eyes) during a surgically induced RD in 2 NHPs using a vehicle solution containing dimethyl sulfoxide, classically used as a chemical solvent. We also performed functional testing of the retina (full-field and multifocal electroretinogram [ERG]).

**Main Outcome Measures:**

Correlation of cone mosaic metrics (cone density, spacing, and regularity) between normal retinae of NHPs and humans, and cone metrics, power spectrum, and ERG wave amplitudes after RD.

**Results:**

Imaging features were very similar in terms of cone reflectivity, cell density, regularity, and spacing values, showing strong positive correlations between NHPs and humans. After RD, AO-FIO revealed several alterations of the cone mosaic slowly recovering during the 3 months after the reattachment, which were not detected functionally by ERG.

**Conclusions:**

These results demonstrate by in vivo AO-FIO imaging the transient structural changes of photoreceptors after an RD in the primate retina. They also provide an interesting illustration of the AO-FIO potential for investigating photoreceptor toxicity during preclinical studies in NHPs with a high translatability to human studies.

**Financial Disclosure(s):**

Proprietary or commercial disclosure may be found after the references.

Novel gene and cell therapies are entering into clinical trials or even becoming commercial.^1^^,^^2^ Many of these clinical trials are targeting the prevention of photoreceptor cell loss, and they require subretinal delivery of viruses or retinal pigment epithelial cells.^3^ Such interventions are occurring before photoreceptor cell loss because they aim at preventing the degenerative process. To investigate the safety of these therapeutic strategies, experiments in nonhuman primates (NHPs), especially Old World monkeys such as cynomolgus macaques, represent the final stage before the clinical study. Indeed, these animals share common structural, functional, and immune retinal features with humans. For instance, the presence of a macula centered by a ‟concaviclivate” foveal pit in the area centralis is a hallmark of these species.^4^ In this regard, the primate model is certainly the most appropriate for understanding and studying human vision and retinal diseases while developing strategies to prevent blindness or restore sight, especially at the macular level.^5^

By adapting the system’s optical properties while performing the acquisition, adaptive optics (AO) imaging provides a lateral resolution of up to 2 μm in the human retina, allowing in vivo direct visualization of photoreceptors, especially cones.^6^ Adaptive optics technology can be combined with several eye imaging approaches, including OCT, scanning laser ophthalmoscopy (SLO), and flood illumination ophthalmoscopy (FIO).^7^ Very few AO devices with regulatory approval are deployed in clinical routine. Adaptive optics FIO (AO-FIO) provides highly sensitive and minimally distorted retinal imaging but with lower resolution than AO-SLO devices.^8^ However, an AO-FIO commercial device is available and has been widely used in clinical studies since 2012.^9^ More recently, features of a wide array of human retinal pathologies (e.g., inherited retinal disease, age-related macular degeneration, diabetic retinopathy) have been described using AO-FIO.^10^^,^^11^ Also, retinae of various species have been studied using this technology.^12^ Despite the relevance of the primate model in the study of retinal disease, and although it may provide a critical bridge between animals and humans in the development of therapeutic strategies, normal AO-FIO findings have not yet been reported in NHPs.

Additionally, because substantial emerging therapies for degenerative retinal disease require subretinal delivery,^3^ it results in short-term induced retinal detachment (RD), usually including the macula. By separating the neuroretina from the underlying retinal pigment epithelium (RPE), RD leads to extensive retinal remodeling, especially in the outer segments (OSs) of photoreceptors and at the apical RPE surface.^13^ Short-term induced RD with balanced salt solution has been studied twice in rhesus monkeys and only once in the macular area.^14^^,^^15^ No study involving NHPs reported RD using dimethyl sulfoxide (DMSO), which is frequently used in the fundamental research field to solubilize therapeutic or toxic agents that are not soluble in water and may therefore have solvent toxicity,^16^ potentially on OSs (especially if delivered in the subretinal space).

In the present study, we investigated normal AO-FIO findings in NHPs and the structural and functional effects of subretinal delivery of DMSO using in vivo AO-FIO and electroretinography.

## Methods

### Animals and Healthy Human Subjects

Four NHPs (*Macaca fascicularis*) were used in this study: 3 5-year-old males (NHPs 1, 2, and 3) and one 15-year-old female (NHP 4). Nonhuman primates 1 and 2 were used for normal and short-term induced RD retinal features during a 4-month follow-up, and NHPs 3 and 4 were used to obtain supplemental normal retinal data and features. Animals were born in captivity and originated from approved suppliers with AAALAC certification (SARL Bioprim, Bazièges, France; Cynologics-Silabe, Niederhausbergen, France). Ethical approvals were obtained from the local ethical committee CETEA n°44 of the Molecular Imaging Research Center and then the French Ministry of Education and Research.

Four healthy human subjects (3 men aged 27, 38, and 58 years and 1 woman aged 25 years) were also included to provide reference values of the human retina. They agreed to participate in this study under proper informed consent. The described data collection was approved by the institutional review board of the Quinze-Vingts National Ophthalmology Hospital and adhered to the tenets of the Declaration of Helsinki.

### Housing, Perioperative Care, and Ethical Approvals in Animals

Nonhuman primate experiments were conducted in the Molecular Imaging Research Center at Fontenay-aux-Roses, France. The animal facility is authorized for animal experimentation on NHPs (n° D 92 032 02) by local authorities and complies with the EU directive requirements regarding the use of animals in research (2010/63/EU). The test facility complies with all the requirements of the EU Directive 2010/63, including housing and care of the animals by authorized trained staff. Animals are subjected to daily controls, which include clinical and behavioral assessments. Trained staff (animal caretakers, veterinarians, and neuroscientists) are responsible for care and housing procedures. Perioperative care, including analgesics, were defined and adjusted by the attending veterinarian and previously validated by the ethical committee.

### Anesthesia

Before data acquisition or surgery, animals were anesthetized with an intramuscular injection of ketamine, 10 mg/kg (Imalgene 1000, Merial) and xylazine 0.5 mg/kg (Rompun 2%, Bayer). Anesthesia was maintained with an intravenous injection of propofol, 1 mL/kg/hour (PropoVet Multidose 10 mg/mL, Zoetis). Then, bilateral mydriasis was obtained with eyedrops of 0.5% tropicamide (Mydriaticum, Théa), and eyelids were kept open using an eyelid speculum. Throughout the procedures, the ocular surface was very regularly wetted with balanced salt solution (Alcon Inc).

### Surgery

Nonhuman primates 1 and 2 each received a subretinal injection in the left eye. A 25-gauge (25G) transconjunctival vitrectomy system (Constellation, Alcon Inc) was used to perform a 100-μL subretinal delivery (subretinal injection canula De Juan/Awh 25G/41G, Synergetics, Inc) of a 0.2% DMSO (D2650, Millipore Sigma) solution in phosphate-buffered saline (14190-094 Life Technologies Europe), without performing vitrectomy. The injection site was set at the branching of the superior temporal vessels, ensuring the bleb to detach the superior hemifovea (Figs S1 and S2 available at www.ophthalmologyscience.org). A full description of the material and the surgical technique used is available in Bertin et al.^17^

### Data Acquisition

#### AO-FIO

The rtx1 (Imagine Eyes), a compact AO-FIO commercially available device, was used to image the photoreceptor layer of the NHPs and healthy human subjects. In the rtx1, retinal images are captured by a low-noise high-resolution charged-coupled device camera (Manta, Allied Vision) while an AO correction system composed of a Shack–Hartmann wavefront sensor (HASO4 first, Imagine Optic) and a deformable mirror (mirao 52-e, Imagine Eyes) work in a closed loop to compensate the aberrations introduced by the eye. Retinal images obtained with the rtx1 have a lateral resolution of a few micrometers over a 4 × 4-degree field of view. The exact lateral resolution has not been studied in NHPs because it depends on several parameters, including axial length and pupillary size, which are different between humans and monkeys. A full technical description of the rtx1 is available in Zacharria et al.^18^ A defocus of +0.50 diopters (corrected by a modified Badal assembly incorporated in the commercial device) was used for NHP imaging. Nonhuman primates were anesthetized during data acquisition because fixation could not be maintained otherwise. Anesthetized animals were laid on the examination table with their head fixed to have their eyes aligned with the camera. Because the eyes cannot be manually moved during the experiments (otherwise, too much astigmatism is induced, preventing proper imaging), the entire table was positioned to explore intended retinal areas (Fig S1B, available at www.ophthalmologyscience.org). The investigator (A.D.) followed the same procedure during each acquisition: (1) locating the fovea with the help of the retinal vascular pattern (Fig S3, available at www.ophthalmologyscience.org) and (2) acquiring 4 images on the superior, inferior, nasal, and temporal sides of the fovea in the central 6°. As far as technically achievable, peripheral retinal images were also captured. The focus (related to the axial depth in micrometers) was set between 0 and +50 to capture the photoreceptor layer as sharp as possible.

Eyedrops of 0.5% tropicamide (Mydriaticum, Théa) were used to obtain mydriasis in human subjects, who were instructed to fixate at 0°, 2°, 4°, and 6° of eccentricity along the nasal, temporal, superior, and inferior retina.

In NHPs and humans, the acquisition consisted of a series of 40 frames over 2 seconds with an exposure time of 10 ms (averaged by the embedded software to produce 1 final image) in a 4 × 4-degree field size, captured at each retinal location mentioned above. Then, 2 types of analysis were performed on each raw picture. (1) The manufacturer commercial software (AODetect 3.0, Imagine Eyes) was used for cone detection, segmentation, and regularity analyses. In a manually set sampling window of 80 × 80 pixels of each acquired retinal area (mentioned below as the region of interest [ROI]), cones were first marked semiautomatically (automatic detection by the software, manually adjusted by A.D.), and the following estimates of density (Voronoi cell density), intercell spacing (the mean distance between each cone photoreceptor and its neighbors), and regularity index were obtained. These metrics are the most commonly reported biomarkers.^11^ Because isodensity lines of the distribution of cones are elongated around the horizontal meridian,^19^ ROIs on the same superior–inferior eccentricity to the fovea were favored. The sampling window for the ROI was placed at the closest location that was free from large vessels to avoid vascular artifacts. The internal software analysis (including the use of Delaunay triangulation and Voronoi diagrams) and the reproducibility of cell density measurements (estimated > 96%) have already been reported.^20^ Density values were computed in cones/degree^2^ and spacing values in arc minutes to remain independent of the axial length, which is different between human and NHP subjects (23–24 mm versus 17–18 mm, respectively). (2) The cone mosaic was also approached by the power spectrum spacing method to describe an additional approach that has been reported in the current literature. The frequency domain allows for the determination of the modal frequency and spacing of the cone mosaic^21^ without the need for segmentation. First, using ImageJ (NIH), a fast Fourier Transform was processed on a 300 × 300-pixel sample of each acquired retinal area (the ROI selection process was identical to the one used for automated analyses) to obtain the corresponding Yellott ring^22^ (Fig S4, available at www.ophthalmologyscience.org). Then, as previously detailed and characterized,^21^ the power spectrum was calculated and expressed as a function of spatial frequency (referred to as the 2-dimensional log_10_-power spectrum) using MATLAB (v.R2022a, The MathWorks, Inc). The resulting correlation plots showed a main peak that corresponds to the cone mosaic regularity and reflectivity (arrow on power spectrum plot in Fig S4, available at www.ophthalmologyscience.org). When needed for a better display of the data, plots were also normalized to spatial frequencies to be independent of retinal eccentricity (Fig S5A, available at www.ophthalmologyscience.org).

Adaptive optics FIO was performed only once in healthy human subjects and in NHPs 3 and 4. In NHPs 1 and 2, AO-FIO was performed before surgery (day 0 = D0); at day 3 (D3), day 15 (D15), and day 30 (D30); and monthly until month 4 (M2, M3, and M4) (Fig S1A, available at www.ophthalmologyscience.org).

#### Additional Anatomical Investigations

Slit-lamp and indirect ophthalmoscopy examinations were frequently performed during the follow-up in NHPs 1 and 2. Fundus color photography was taken with the Smartscope camera (Optomed, Oy). Spectral-domain OCT high definition horizontal and vertical B-scans through the foveal location and the injection point were also collected at the time of AO-FIO imaging using the Spectralis HRA+OCT imaging system (Heidelberg Engineering), mainly to assess reattachment. We also acquired 30- and 55-degree multicolor, infrared reflectance, short-wave, and near-infrared fundus autofluorescence (Fig S2).

#### Functional Testing

Full-field electroretinograms (ffERGs) and multifocal electroretinograms (mfERGs) were performed in NHPs 1 and 2 in accordance with the International Society for Clinical Electrophysiology of Vision guidelines^23^^,^^24^ by using the RETI-map (Roland Consult). A combination of SLO with mfERG allowed simultaneous infrared laser monitoring, and the embedded software (Roland Consult Fundus EyeTracker) detected any eye movement artifacts during examination. The stimulation pattern isolated 37 hexagonal retinal areas in a 30-degree diameter field, with hexagon 19 placed at the foveal level. Recording was performed at a 59.8-Hz frame rate with 1-kHz (983.5 μs) sampling. ERG-Jet Electrode RC (RC 1000-530-330-D, Fabrinal Eye Care) for the ffERG and clip-connector electrodes (ref. PEAV, SIEM Bio-Médicale) for the mfERG were used after applying local anesthesia (oxybuprocaine eyedrops, 1.6 mg/0.4 mL, Théa).

### Statistical Analyses

Differences in cone density, regularity, and spacing values between NHPs and humans were assessed using a 2-tailed *t* test, and the interspecies correlations between these values were estimated using Pearson correlation coefficients. Electroretinogram wave amplitudes were compared between baseline and consecutive examinations in NHPs 1 and 2 using repeated measures 2-way analysis of variance with the Geisser–Greenhouse correction. A *P* value < 0.05 was considered statistically significant. Analyses and charts were created using GraphPad Prism 8.4.0 (GraphPad Software Inc).

### Color Palette

The color palette in graphs and images throughout this article has been altered to accommodate colorblind readers.

## Results

### Qualitative AO Flood Illuminated Features in Normal Retinae

**Figure 6:**
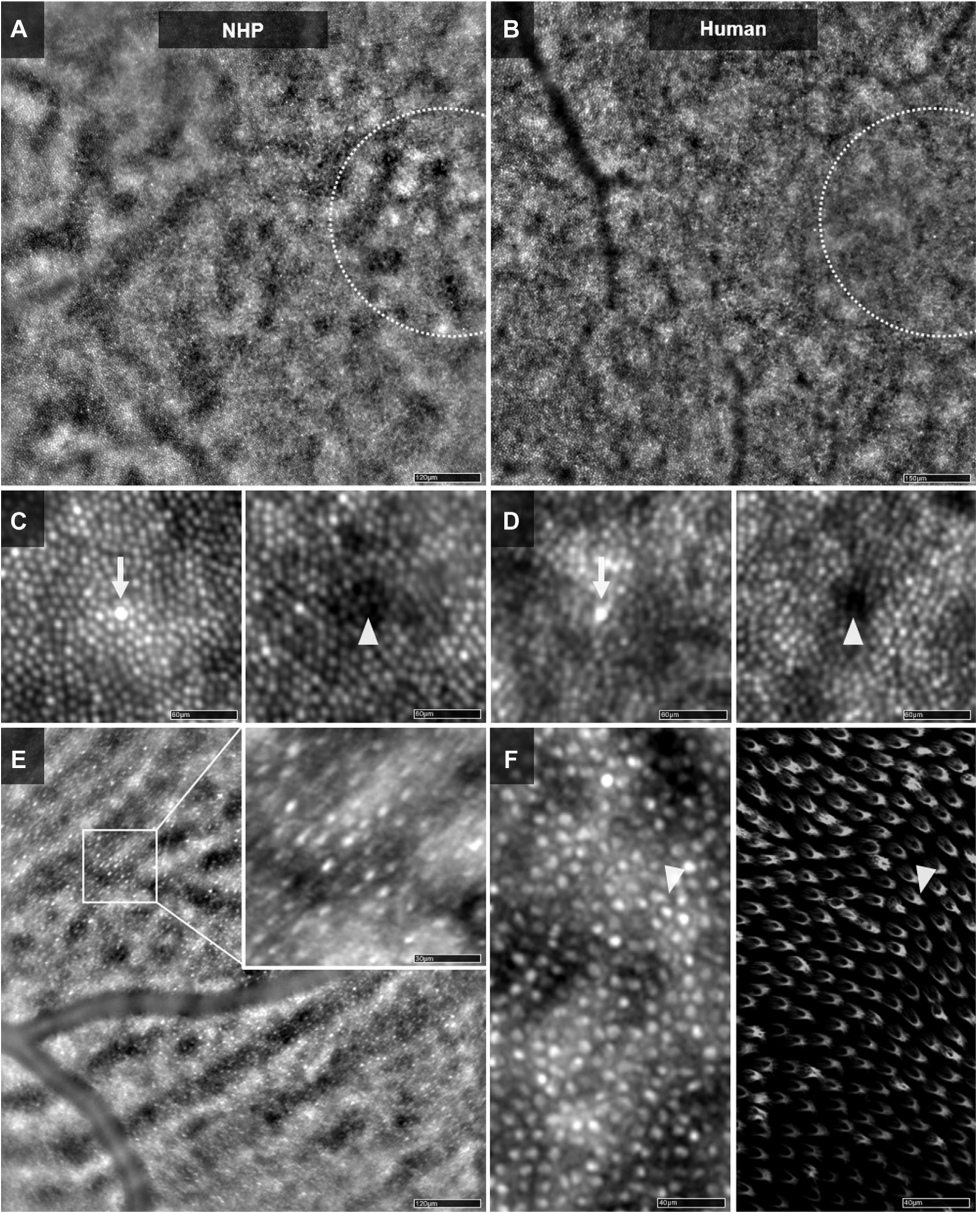
Adaptive optics flood illumination (AO-FIO) main features in nonhuman primate (NHP) compared with human. **A–B**, 4 × 4-degree field centered on the temporal side of the fovea in an NHP (**A**) and human (**B**) at the photoreceptor layer (horizontal eccentricity = 1°). The foveal area (delimited by the dotted circles) is hallmarked by hyperreflective clumps of cones better seen in the NHP (**A**). **C–D**, Hyperreflective (arrows) and darker (arrowheads) cones are shown in the para- and perifoveal areas both for the NHP (**C**) and human (**D**). **E**, Peripheral (20-degree) retinal AO-FIO imaging in an NHP showing the slender aspect of cones, with their expected outer segments (OSs). **F**, Comparative AO-FIO imaging at 6° of eccentricity (left) and ex vivo immunofluorescence histological examination after cone labeling (peanut agglutinin lectin, right) in the NHP retina showing the matching aspect of the OS, which can be spotted in the intercone spaces in both AO-FIO and histology (arrowheads). Scale bars: A: 120 μm, B: 150 μm, C–D: 60 μm, E: 30, and 160 μm, F: 40 μm.

Figure 6 illustrates the comparative observations of the human and NHP retinae with the AO-FIO technology in 4 × 4-degree retinal fields. The macular area is hallmarked by highly hyperreflective cones either clustered in heterogeneous (fovea) or isolated (parafovea) bunches (Fig 6A–D). As previously reported in humans,^3^ bright and dark cones were observed surrounded by less defined rods in NHPs (Fig 6C). The corresponding dark spaces between cones were occasionally filled seemingly by OSs and rods (Fig 6E–F). At this level, the cone mosaic appeared more homogeneous than in humans (Fig 6C–D). In contrast, the peripheral NHP retina showed irregularly shaped cones with irregular shapes of OSs (Fig 6E). Additional AO-FIO images and features in NHPs are available in Figure S7 (available at www.ophthalmologyscience.org).

### Comparative Quantitative AO Features

**Figure 8:**
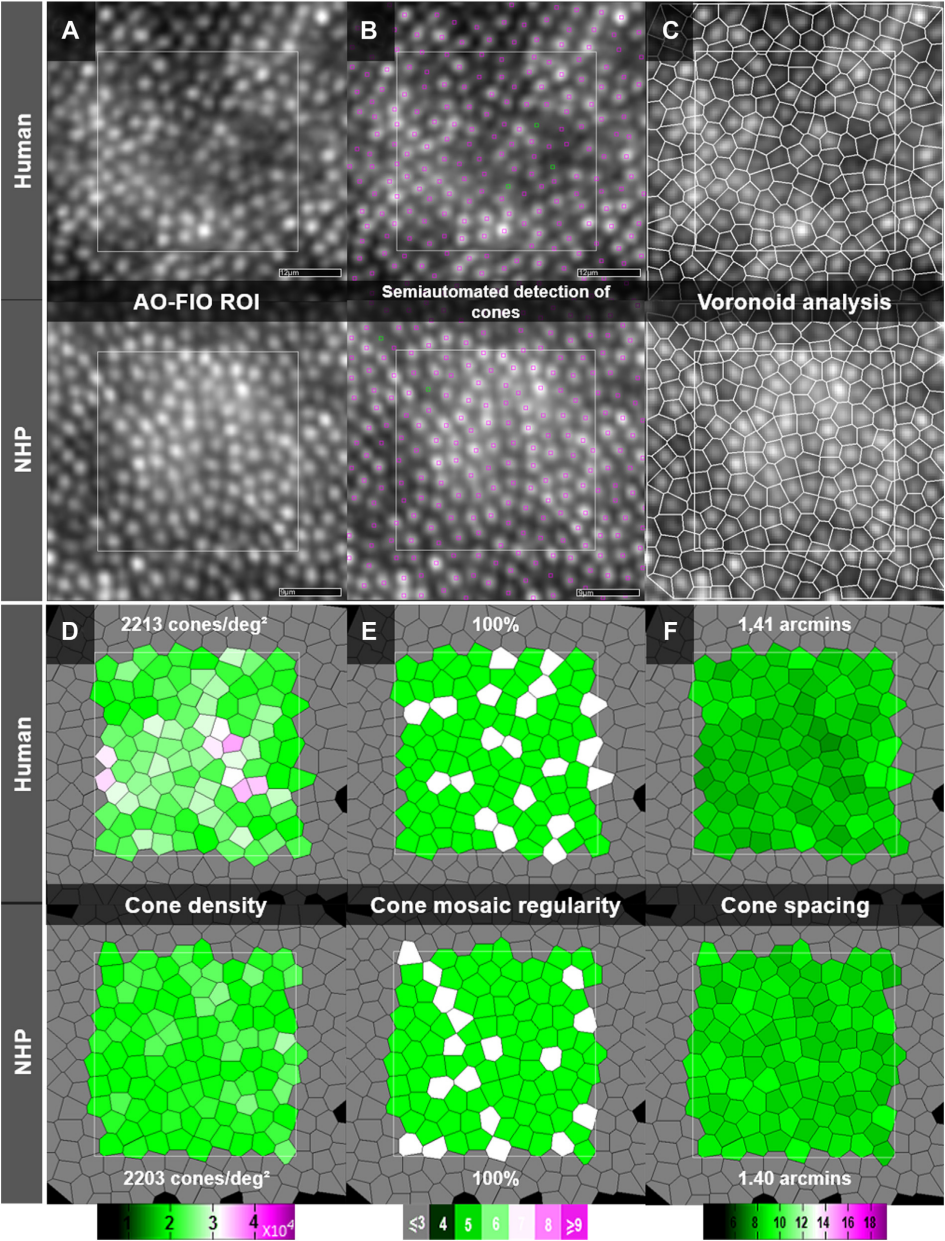
Cone mosaic analyses on adaptive optics flood illumination (AO-FIO) imaging in human (top) and nonhuman primate (NHP; bottom) (parafoveal area, horizontal eccentricity = 2°). A region of interest (ROI) of 80 × 80 pixels (**A**) is semiautomatically segmented (**B**). Cones automatically segmented by the embedded software are marked in red; those manually corrected appear in green. The following Voronoi analysis allows us to determine cone density (**D**), regularity (**E**), and spacing (**F**). Note that the given values are comparable between humans and NHPs. Human scale bars: 12 μm. Nonhuman primate scale bars: 9 μm. Colored scales: D: estimated cone density in cones/mm^2^, E: estimated cone regularity with the number of neighboring cells, F: estimated cone spacing in microns.

**Figure 9:**
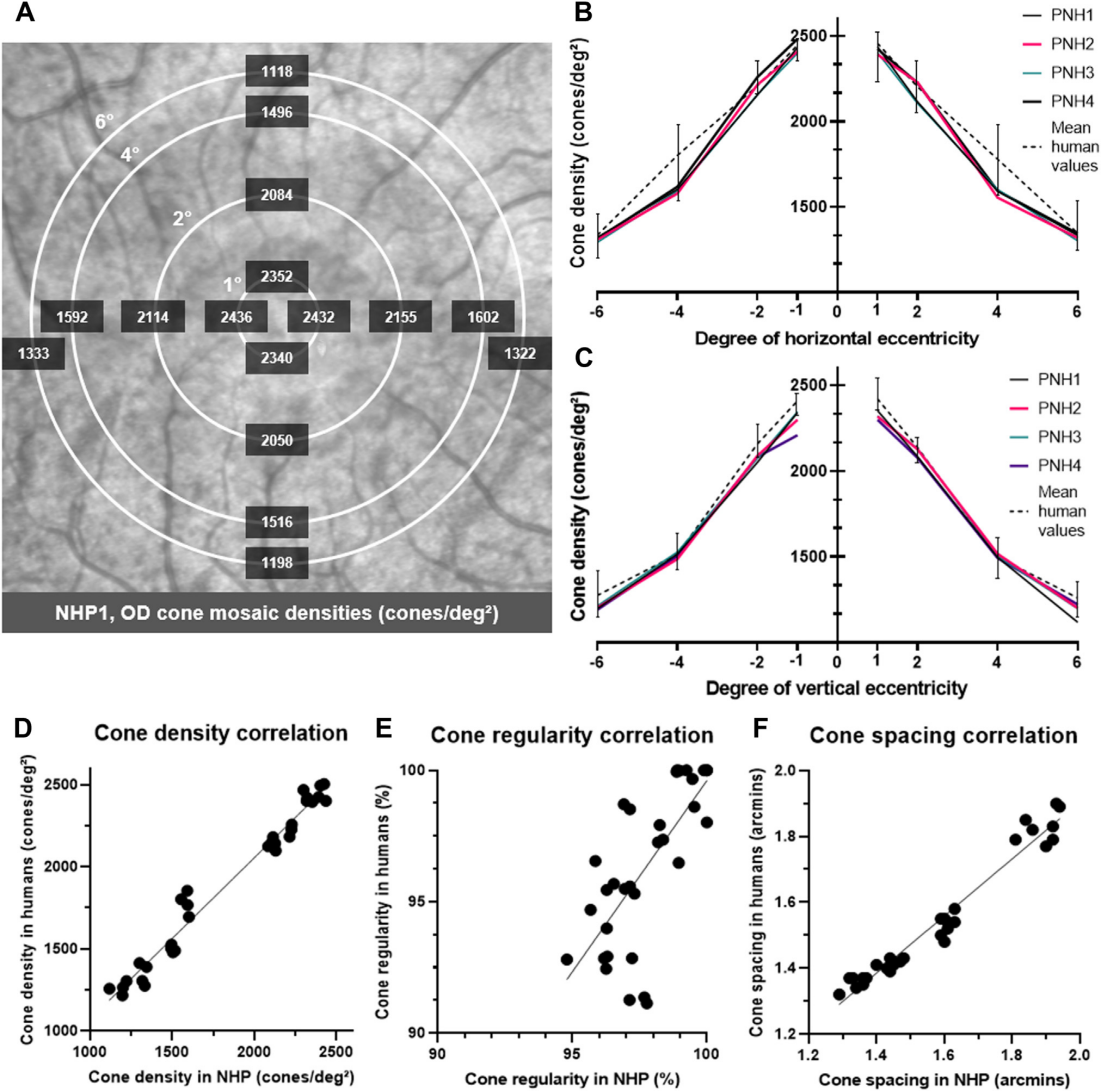
Cone mosaic metrics in human and nonhuman primates (NHPs**). A**, Sample of density values computed in NHP 1 in the right eye (OD), in cones/deg^2^ along each temporal (left), superior (top), nasal (right), and inferior (bottom) eccentricity. **B–C**, Mean human and individual NHP cone density values (in cones/deg^2^) according to horizontal (**B**) and vertical (**C**) eccentricity (in degrees, from nasal to temporal and inferior to superior, respectively; negative eccentricities are toward the optic disc and the inferior retina for the horizontal and vertical axes, respectively). Error bars show standard deviation of human values. **D–F**, Correlation plots of density (**D**), regularity (**E**), and spacing (**F**) values between humans and NHPs (x-axis and y-axis are equal). Simple linear regressions are shown (black curves, slopes = 0.98, 0.69, and 0.87, respectively). Scale bar: 200 μm.

Samples of cone mosaic analyses at 2° of eccentricity on AO-FIO imaging in a human and an NHP are presented in Figure 8. Mean cone mosaic density, regularity, and spacing values computed in NHPs and humans are presented in Tables 1 and 2 (individual values are available in Tables S3–S6, available at www.ophthalmology.org). Along the horizontal axis, cone density values showed a shallower decreasing slope in humans at 4° of eccentricity. Otherwise, cone density matched between humans and NHPs (Fig 9B–C). Cone mosaic regularity reached 100% in the parafoveal area (4- and 6-degree) in both NHPs and humans. Correlations between individual cone density, regularity, and spacing values of NHPs and humans are shown in Figure 9D–F. A significant positive correlation was found in all 3 cases (*r* = 0.99; 95% confidence interval [CI], 0.97–0.99; *P* < 0.0001; *r* = 0.71; 95% CI, 0.48–0.85; *P* < 0.0001; and *r* = 0.98; 95% CI, 0.96-0.99; *P* < 0.0001, respectively).

**Table 1:** Mean Cone Mosaic Metrics Values in NHPs and Humans along the Horizontal Axis (toward the Temporal Retina)

Values	Horizontal Eccentricity (Degrees)	NHPs (Mean Values ± SD)	Humans (Mean Values ± SD)	*P*
Cone density (cones/deg^2^)	1°	2413 ± 20	2456 ± 51	0.1729
	2°	2168 ± 68	2202 ± 49	0.4481
	4°	1586 ± 21	1779 ± 67	**0.0015**
	6°	1324 ± 51	1346 ± 66	0.5537
Cone mosaic regularity (%)	1°	96.49 ± 1.29	91.91 ± 0.84	**<0.0001**
	2°	98.53 ± 1.29	97.78 ± 0.94	0.3838
	4°	99.44 ± 0.59	99.99 ± 0.02	0.1154
	6°	96.45 ± 0.68	95.28 ± 0.42	**0.0261**
Cone spacing (arcmin)	1°	1.33 ± 0.03	1.35 ± 0.02	0.4372
	2°	1.43 ± 0.02	1.41 ± 0.01	0.2110
	4°	1.61 ± 0.02	1.51 ± 0.03	**0.0007**
	6°	1.85 ± 0.04	1.81 ± 0.04	0.1310

Statistically significant *P* values are shown in bold. NHP = nonhuman primate; SD = standard deviation.

**Table 2:** Mean Cone Mosaic Metrics Values in NHPs and Humans along the Vertical Axis (toward the Superior Retina)

Values	Vertical Eccentricity (Degrees)	NHPs (Mean Values ± SD)	Humans (Mean Values ± SD)	*P*
Cone density (cones/deg^2^)	1°	2323 ± 22	2421 ± 33	**0.0025**
	2°	2138 ± 55	2137 ± 36	0.9648
	4°	1503 ± 11	1499 ± 21	0.7741
	6°	1185 ± 46	1260 ± 36	**0.0399**
Cone mosaic regularity (%)	1°	96.85 ± 0.73	92.49 ± 0.75	**0.0002**
	2°	98.31 ± 0.98	97.94 ± 0.73	0.5646
	4°	99.41 ± 0.46	99.92 ± 0.17	0.0812
	6°	96.56 ± 0.59	95.40 ± 1.06	0.1053
Cone spacing (arcmin)	1°	1.35 ± 0.02	1.37 ± 0.01	0.3437
	2°	1.46 ± 0.02	1.42 ± 0.02	**0.0289**
	4°	1.61 ± 0.02	1.56 ± 0.02	**0.0050**
	6°	1.93 ± 0.01	1.85 ± 0.05	**0.0294**

Statistically significant *P* values are shown in bold. NHP = nonhuman primate; SD = standard deviation.

Peripheral retinal imaging in NHPs showed cone density of 520 cones/deg^2^, 100% regularity, and 2.89 arcmins spacing at a vertical eccentricity of 10°, and a cone density of 400 cones/deg^2^, 89% regularity, and 3.50 arcmins spacing at a vertical eccentricity of 20°.

Samples of AO-FIO imaging of the photoreceptor layer corresponding Yellott’s rings and power spectrum are provided in Figure S4 (available at www.ophthalmologyscience.org) to show that patterns are very similar between humans and NHPs in each eccentricity.

### AO Flood Illuminated Outcomes in Short-term Induced RD

**Figure 10:**
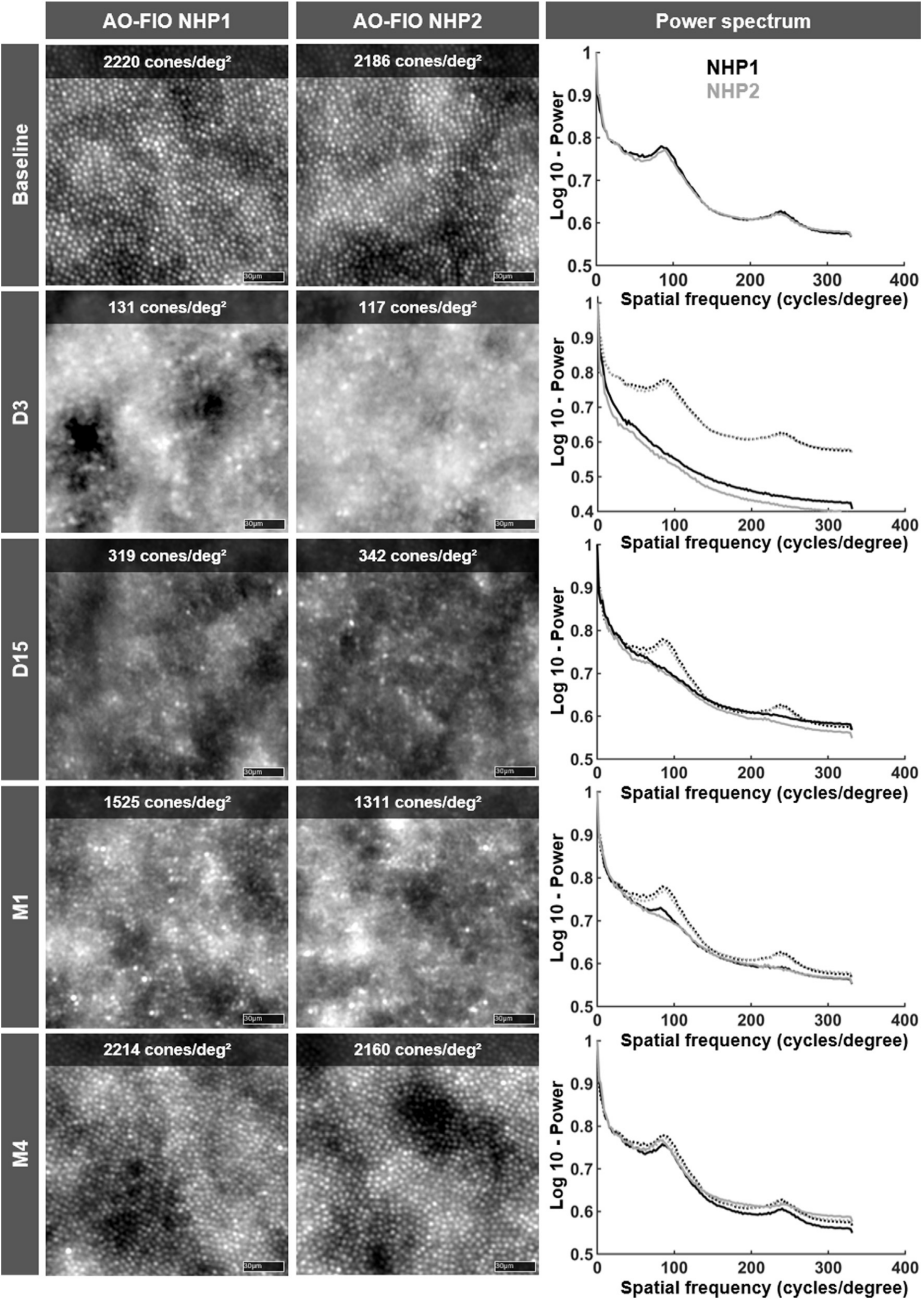
Adaptive optics flood illumination ophthalmoscopy (AO-FIO) imaging and corresponding power spectrum plots after short-term detached photoreceptor macular layer with DMSO (horizontal eccentricity = 2°) in NHPs 1 and 2. Semiautomated count of cone mosaic density is provided on AO-FIO imaging. Note that AO-FIO shows very heterogeneous cone aspects between D3 and M1, with "brilliant cones" previously described in inherited retinal diseases. The power spectrum shows either an absent (D3) or a much shallower cone mosaic peak (D15 and M1) with an almost reached baseline peak at M4 postreattachment in both NHPs 1 and 2. Scale bars: 30 μm. NHP = nonhuman primate.

Figure 10 illustrates the AO-FIO imaging and power spectrum analyses after short-term RD in the macula at 2° of temporal eccentricity area with a 0.2% DMSO solution. The edge of the detached area and the nondetached area imaged at day 15 shows a clear alteration of the cone mosaic (Fig 10). Although concomitant OCT suggested complete retinal reattachment at day 3 (Fig S2), power spectrum and cone reflectivity in AO-FIO appeared compromised until M4 (Fig 10). A progressive return to the baseline spatial frequency peak was observed during the 4-month follow-up after induced RD (Fig 10 and Fig S5).

It has to be noted that short-term induced RD using DMSO in NHPs seems to result in demarcation lines still visible on autofluorescence and infrared retinal imaging after several months (Fig S2), which is not expected after an acute RD in humans.

### Functional Outcomes in Short-term Induced RD

**Figure 11:**
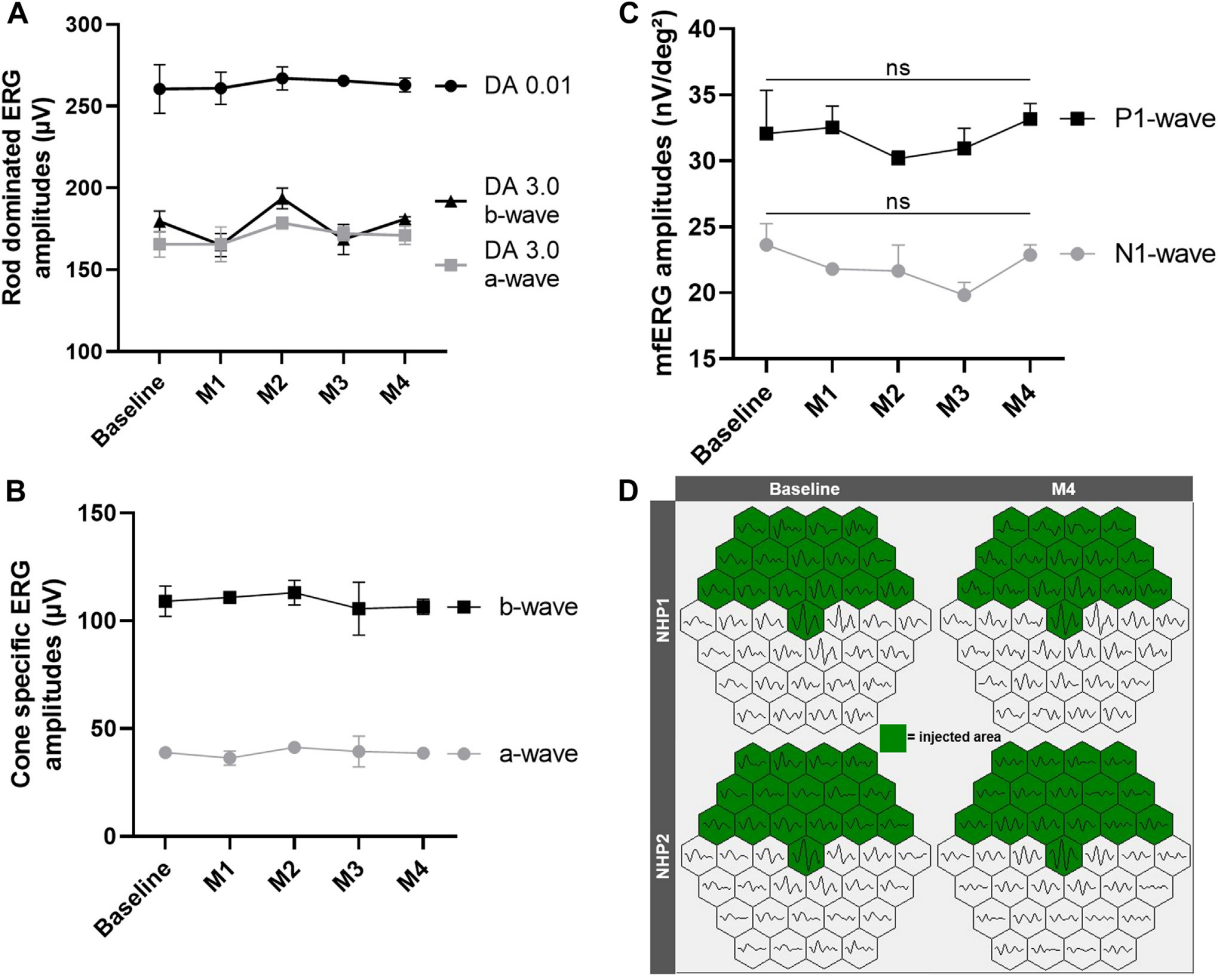
Functional in vivo testing before short-term induced retinal detachment (RD) and after retinal reattachment in NHPs 1 and 2. A-B, Dark-adapted (DA in **A**) and light-adapted (LA in **B**) full-field electroretinograms showing no significant changes in amplitudes before and after RD (2-way analysis of variance *P* = 0.1320 and *P* = 0.5710 for rod-dominated and cone-specific ERG, respectively). **C**, Multifocal electroretinogram (mfERG) amplitudes of N1-wave (gray plot) and P1-wave (black plot) in the detached hexagons showing no significant changes in both waves’ amplitudes before and after RD (2-way analysis of variance *P* = 0.51 for P1-wave and *P* = 0.21 for N1-wave). ERG = electroretinogram; NHP = nonhuman primate.

Rod- and cone-specific ffERG and mfERG amplitudes were recorded in parallel at baseline and during the 4-month follow-up after retinal reattachment (Fig 11). Dark-adapted and light-adapted ffERG amplitudes did not show any disturbance during the post-RD follow-up (Fig 11A, B, *P* = 0.1320 and *P* = 0.5710 for rod-dominated and cone-specific electroretinograms, respectively), which is consistent with the induced RD involving only a small proportion of the whole retina. Analyses of mfERG with the quantification of the N1- and P1-wave amplitudes in each hexagon within the detached area showed no statistically significant differences during the follow-up (Fig 11C, *P* = 0.51 for P1-wave and *P* = 0.21 for N1-wave). Samples of mfERG recordings are shown in Figure 11D (green hexagons were within the detached area). These data suggest that changes observed on retinal AO imaging cannot be detected on mfERG.

## Discussion

We examined by AO-FIO the photoreceptor layer in 4 NHPs showing that the cone mosaic exhibits similar patterns and metrics when compared with 4 humans. We further demonstrate that the AO-FIO provides evidence of photoreceptor structural alterations despite no functional change after a subretinal detachment. To the best of our knowledge, this is the first study to describe the NHP photoreceptors by AO-FIO with normal findings. In 2014, one study reported measurements of cone density in Japanese macaques (*Macaca fuscata*) with drusenoid type retinal alterations, thus without reporting normal features.^25^

Adaptive optics FIO is one of the most used eye imaging systems based on AO technology to examine photoreceptors in clinical practice. This technology may therefore be an interesting tool to adopt in preclinical studies to enhance translatability of the data. Other AO devices have been previously used in NHPs mostly to investigate photoreceptor functionality under specified conditions, such as fluorescence AO scanning light ophthalmoscopy.^26^ However, these tools are not currently usable in clinical practice because they are custom-made and designed only for laboratory purposes. Moreover, data generated by the differing AO-based imaging systems are not comparable, which greatly limits the current use of devices that are not suitable for clinical practice, thus restricting potential bridges with preclinical studies.

Adaptive optics FIO seems to provide reliable cone mosaic metrics values. Indeed, we found very similar values in the human subjects to those previously reported in larger healthy populations.^20^ Moreover, they are in accordance with measurements obtained from human whole-mounted retinae.^19^ In NHPs, our findings are also in agreement with previously reported histological studies conducted on adult *Macaca menestrina*: as described here, the mean cone density values were decreasing with eccentricity more greatly along the vertical retinal axis than the horizontal one.^27^ Thus, it can be assumed that this imaging device and its related analysis software intended for human clinical practice can still be used for cone mosaic assessment in NHPs, allowing the investigation of in vivo features at the photoreceptor level in a relevant model of human retinal disease.

**Figure 12:**
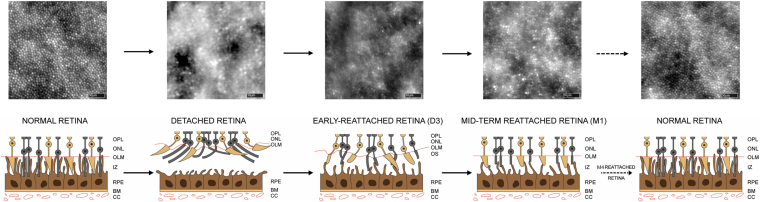
Adaptive optics flood illumination ophthalmoscopy (AO-FIO) images with photoreceptor and retinal pigment epithelium (RPE) schematic representation after short-term induced retinal detachment (RD) (based on this study results and preexisting literature). Total disruption of the interdigitating zone (IZ) is caused by RD, leading to floating outer segments (OSs) in the subretinal fluid. Early-reattached retina shows emerging interactions between OSs and the RPE’s apical surface. Misaligned photoreceptors perikaryons and OSs may explain AO-FIO heterogeneous aspect in early-reattached and mid-term reattached retinae. A return to the normal aspect is allowed by fully recovered OS length and alignment, as well as the recovery of OS–RPE interactions. Scale bars: 30 μm. BM = Bruch’s membrane; CC = choriocapillaris; OLM = outer limiting membrane; ONL = outer nuclear layer; OPL = outer plexiform layer.

Follow-up AO-FIO imaging after rhegmatogenous RD has been previously reported in humans for clinical purposes.^28^ However, the pathophysiology of this type of RD, its duration, and the subretinal fluid composition are very different from those induced in the course of fundamental research or subretinal therapy delivery.^29^ We attempted to characterize postreattachment reflectivity of cones in AO-FIO after a short-term (< 3 days) induced RD using DMSO. We showed that the cone mosaic aspect almost recovers only after 4 months using cone metrics and the power spectrum method despite anatomical reattachment 3 days after the RD. The altered cone mosaic in AO-FIO during the 4-month period after reattachment can be explained by several factors. Studies on various animal models (rodents and cats) showed early shortening of the OS (inner segments remain morphologically unchanged) with postreattachment apparent recovery and reestablishment of the RPE-photoreceptor interface.^30^ In rhesus monkeys, OS mean length has been shown to recover totally after 5 months of reattachment after a short-term induced RD with balanced salt solution.^14^^,^^15^ Considering our results and those previously reported, we propose a schematic representation of photoreceptors and RPE alterations after short-term induced RD (Fig 12). We suggest that the RD could induce OS disorganization with a subsequent realignment according to the Stiles–Crawford effect taking several months to fully occur.^31^ Furthermore, the demarcation lines well seen along the follow-up in Figure S2 are possibly related to the composition of the injected solution, which may predominantly affect the edges of the detached retina for solubility purposes.

Additionally, early triggered cellular events (1–3 days after RD) that have been shown to persist after reattachment, such as caspase-dependent apoptosis, necrosis, inflammatory signals from suffering photoreceptors,^32^ and modified opsin redistribution,^30^ could explain why the retina may not anatomically be completely restored soon after an induced RD, however short, especially if the RD involved the macula.^15^ These cellular events lead to subcellular structural changes of photoreceptor that can also, before OS shortening, modify their reflectance in AO-FIO, thus enabling the tool to detect subtle alterations impacting their functionality.^33^ However, we further demonstrated that AO-FIO provides evidence of photoreceptor structural alterations despite no functional changes at 1 month after reattachment. Previously cited subcellular changes, such as modified opsin redistribution, may support preserved function in the case of structural alterations (such as OS shortening), highlighting the need for structural and functional testing in photoreceptor assessment. Moreover, the Stiles–Crawford method of looking at cones that are oriented off axis could help to assess more exactly the presence of photoreceptors.^34^

Interestingly, AO-FIO cone aspects in days 3 and 15 after subretinal injection, while retina is fully reapplied at this time, showed a similar appearance to what has been described as “brilliant cones” in inherited retinal dystrophies.^35^ This supports the assumption that “invisible” or “dysflective cones”^33^ that have altered functionality or structure (e.g., shortening of OSs) are still present and thus potential candidates for retinal therapy. In this regard, noninvasive functional assessment coupled with imaging (photostimulation) could be an easy way to assess the status of those photoreceptors.^36^

Considering that such cones are found in AO-FIO after a single short-term induced RD and the subcellular and cellular changes previously mentioned, we can suppose that these elements could be responsible for some adverse effects observed in human pathology after subretinal delivery of approved retinal gene therapy,^37^ for which recent preclinical and clinical studies did not involve the use of AO technologies to assess safety and tolerance.^1^ In this regard, AO-FIO can be an important tool for preclinical and clinical trials on NHPs and humans. Indeed, some very prevalent macular diseases such as age-related macular degeneration or central serous chorioretinopathy commonly result in macular subretinal fluid formation, which we can attempt to model in NHPs with subretinal delivery.

Finally, we acknowledge some limitations to this study. First of all, the number of human subjects included is largely below the sample sizes compared with previous studies reporting AO-FIO findings in humans. However, we did not necessarily have to include more participants because the 1:1 matching with NHPs seemed adequate. In addition, we did include 4 control NHPs, which is a suitable sample, despite the fact that NHP 4 was 15 years old and that changes in cone structure over a 10-year age difference are not well established in NHPs. However, the raw data provided in the supplementary tables show no major changes in cone metrics in NHP 4 in comparison with the younger ones. Also, we did not provide histological data that could be interesting for histoclinical correlation of the results observed in AO-FIO. We wanted to keep the NHPs alive to be able to study more extensively the long-term toxic effects of RD because long-term toxic effects have been associated with RD in subretinal gene therapy.^37^ No such toxic effect was seen here, perhaps because of shorter analysis and different fluid application (no use of viruses). Another recent case series reported photoreceptor AO-SLO outcomes after a subretinal delivery of a viral vector for the treatment of choroideremia, showing a preserved cone mosaic at 1 month after delivery.^38^ Although it supports the fact that AO can be used to assess subretinal gene augmentation toxicity by subretinal injection, the follow-up after an induced RD should be more extended in those cases because we observed changes up to 4 months in primates only with DMSO. Further studies are needed to define if RD leads to such secondary long-term toxicity as suggested by the speed of the subretinal fluid injection.^39^^,^^40^

## Supplementary Data

Figure S1 Schematic illustrations of methods employed in nonhuman primates (NHPs). **A**, In NHP1 and 2, the follow-up consisted in retinal anatomical investigations at baseline, day 0, 3, 15, months 1, 2, 3, and 4. Functional testing was performed at baseline, months 1, 2, 3, and 4. **B**, In NHPs, retinal imaging with the rtx1 was performed using an adjustable electric table allowing to tilt the NHP. The device does not allow rotating the objective, which is fixed on the horizontal and vertical axis. **C**, NHP1 and 2 underwent a subretinal injection using a pars plana vitrectomy approach and a subretinal injection cannula. The injection point was set at the division of the upper temporal retinal vessels. The resulting bleb detached the superior hemifovea (see also Fig S2 available at www.ophthalmologyscience.org).

Figure S2 Sample of multimodal 55-degree field retinal imaging of the left eye of nonhuman primate 1 (NHP1) showing short-term induced retinal detachment using dimethyl sulfoxide (DMSO) and follow-up examinations. **A**, Infrared (IR) imaging (left) showing OCT (right) b-scan slicing spots (green line). OCT confirmed subretinal delivery of DMSO with detachment of the superior hemifovea. Reattachment was complete at D3. **B**, Short-wave autofluorescence showing early hyperautofluorescence at the injection site, heterogeneous autofluorescence in the detached area, and hypoautofluorescence annulus at the inner edge of the bleb. **C**, Near-IR autofluorescence showing early hyperautofluorescence at the foveal location (D3), progressively returning to a normal inner aspect with persisting demarcation line (M4). Scale bars: 200 μm.

Figure S3 Sample of adaptive optics flood illumination ophthalmoscopy (AO-FIO) in nonhuman primate 2 (NHP2) showing the retinal vasculature landmarks allowing to follow-up changes after retinal detachment (RD) at the same location. **A**, Infrared imaging of the left eye before induced RD in NHP2. AO-FIO imaging samples are overlaid to show that the vasculature of the primate is well recognized and can ensure repeatability. **B–D**, Sample of AO-FIO imaging at 2° of superior eccentricity at baseline (**B**), 2 months (**C**), and 4 months (**D**) after short-term induced RD. The same recognizable vessel is spotted (white arrow). The superior foveal area is shown (white star). Scale bars: A: 200 μm, B–D: 120 μm.

Figure S4 Sample of adaptive optics flood illumination imaging (AO-FIO) of the photoreceptor layer in nonhuman primate 1 (NHP1) and human 1 according to horizontal eccentricity (away from the optic disk) with corresponding Yellott’s ring and power spectrum. Note that the Yellott’s ring gets smaller and thinner with eccentricity in both the NHP and human. Similarly, the cone mosaic peak (black arrow) on the power spectrum sets at decreasing spatial frequencies with eccentricity. Interestingly, the peak looks sharper in the NHP in all eccentricities, suggesting that cone reflectivity is more homogeneous in NHP than in human. Despite that the power spectrum plots are almost totally overlapping in the 2 species, a likely artifactual shift between spatial frequencies at peak can be observed between 2- and 4-degrees. Scale bars: NHP: 30 μm, human: 40 μm.

Figure S5 Sample of power spectrum changes after short-term retinal detachment (RD) in nonhuman primate 1 (NHP1). **A**, Merged power spectrum plots of follow-up retinal adaptive optics flood illumination (AO-FIO) 300 × 300 pixel images at 2-degrees of temporal eccentricity in NHP1. Note the progressive normalization of the power spectrum. The black dashed line indicates the peak spatial frequency at baseline, also reached at M4. **B**, Sample 4 × 4-degree AO-FIO imaging of the photoreceptor layer at the edge of the detached area 15 days after the reattachment and the corresponding power spectrum in 300 × 300 pixels windows (gray window and plot: detached area, black window and plot: nondetached area). The dashed white line highlights the borderline between the detached and nondetached areas. The dark line on the power spectrum plot indicates the peak spatial frequency at the nondetached area window. Scale bar: 120 μm.

Figure S7 Additional adaptive optics flood illumination (AO-FIO) photoreceptor layer imaging and features in nonhuman primates (NHPs). **A**, AO-FIO photoreceptor layer imaging in the right eye of NHP1 at the upper temporal branches division (8 x 4-degree field, montage using i2K retina AO, DualAlign LCC, Troy, NY). **B**, Corresponding AO-FIO field showing the ‘edge effects’ of different sized vessels: totally masked cones at the edge of major vessels (arrows) and partially masked cones at the edge of thinner vessels (arrowhead). **C**, AO-FIO imaging of the temporal side of the optic disk rim (left eye), a perioptic 50-60 μm rim free of distinguishable cones. Scale bars: A–C: 120 μm, highlighted area in C: 30 μm.
