# Supplementary material for: Adaptive Optics Flood Illumination Ophthalmoscopy in Nonhuman Primates: Findings in Normal and Short-term Induced Detached Retinae
Source: Ophthalmol Sci. 2023 Apr 20;3(4):100316. doi: 10.1016/j.xops.2023.100316 (PMC10238594; doi:10.1016/j.xops.2023.100316)
Supplement: Figure S1 — Schematic illustrations of methods employed in nonhuman primates (NHPs). A, In NHP1 and 2, the follow-up consisted in retinal anatomical investigations at baseline, day 0, 3, 15, months 1, 2, 3, and 4. Functional testing was performed at baseline, months 1, 2, 3, and 4. B, In NHPs, retinal imaging with the rtx1 was performed using an adjustable electric table allowing to tilt the NHP. The device does not allow rotating the objective, which is fixed on the horizontal and vertical axis. C, NHP1 and 2 underwent a subretinal injection using a pars plana vitrectomy approach and a subretinal injection cannula. The injection point was set at the division of the upper temporal retinal vessels. The resulting bleb detached the superior hemifovea (see also Fig S2 available at www.ophthalmologyscience.org). [file mmc1.pdf]

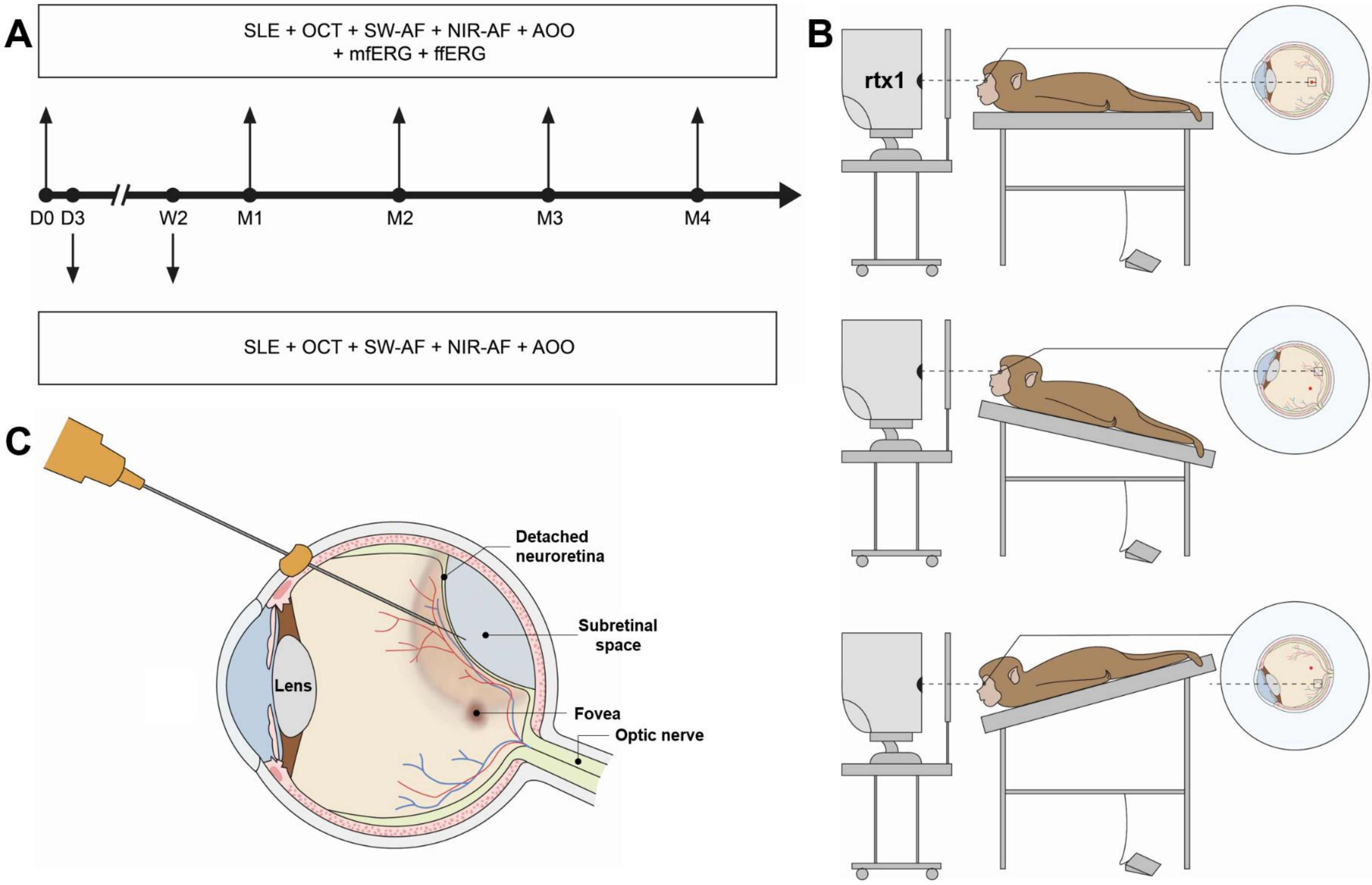

Figure S1. Schematic illustrations of methods employed in non-human primates (NHP). **A.** In NHP1 and 2, the follow up consisted in retinal anatomical investigations at baseline, day 0, 3, 15, months 1, 2, 3 and 4. Functional testing was performed at baseline, months 1, 2, 3 and 4. **B.** In NHP, retinal imaging with the rtx1 was performed using an adjustable electric table allowing to tilt the NHP. The device does not allow rotating the objective, which is fixed on the horizontal and vertical axis. **C.** NHP1 and 2 underwent a subretinal injection using a *pars plana* vitrectomy approach and a subretinal injection cannula. The injection point was set at the division of the upper temporal retinal vessels. The resulting bleb detached the superior hemifovea (see also figure S2 available at https://www.aaojournal.org).
