# Supplementary material for: Adaptive Optics Flood Illumination Ophthalmoscopy in Nonhuman Primates: Findings in Normal and Short-term Induced Detached Retinae
Source: Ophthalmol Sci. 2023 Apr 20;3(4):100316. doi: 10.1016/j.xops.2023.100316 (PMC10238594; doi:10.1016/j.xops.2023.100316)
Supplement: Figure S2 — Sample of multimodal 55-degree field retinal imaging of the left eye of nonhuman primate 1 (NHP1) showing short-term induced retinal detachment using dimethyl sulfoxide (DMSO) and follow-up examinations. A, Infrared (IR) imaging (left) showing OCT (right) b-scan slicing spots (green line). OCT confirmed subretinal delivery of DMSO with detachment of the superior hemifovea. Reattachment was complete at D3. B, Short-wave autofluorescence showing early hyperautofluorescence at the injection site, heterogeneous autofluorescence in the detached area, and hypoautofluorescence annulus at the inner edge of the bleb. C, Near-IR autofluorescence showing early hyperautofluorescence at the foveal location (D3), progressively returning to a normal inner aspect with persisting demarcation line (M4). Scale bars: 200 μm. [file mmc2.pdf]

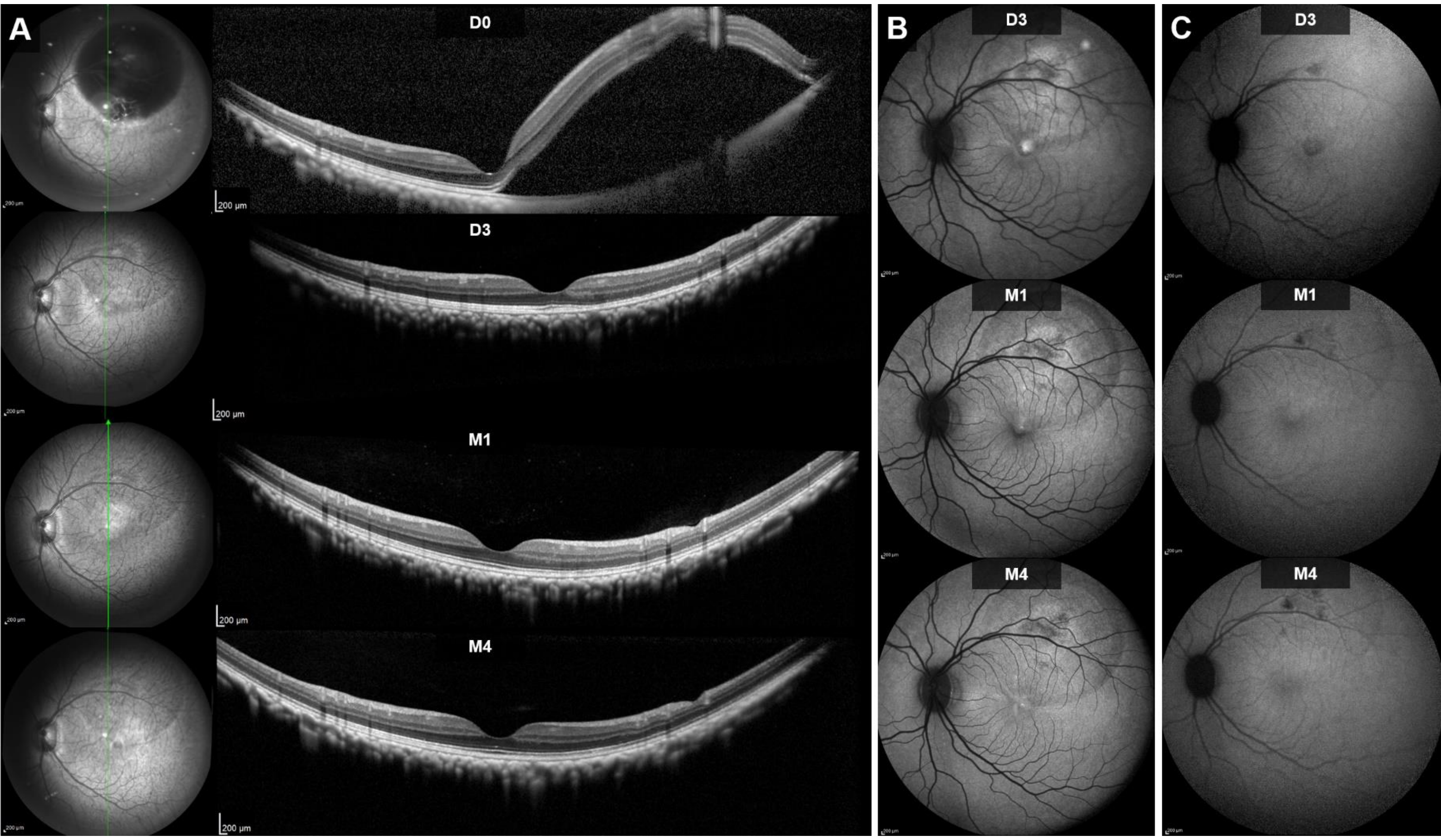

Figure S2. Sample of multimodal 55-degree field retinal imaging of the left eye of non-human primate 1 (NHP1) showing short-term induced retinal detachment using dimethyl sulfoxide (DMSO) and follow up examinations. A. Infrared (IR) imaging (left) showing optical coherence tomography (OCT, right) b-scan slicing spots (green line). OCT confirmed subretinal delivery of DMSO with detachment of the superior hemifovea. Reattachment was complete at D3. **B.** Short-wave autofluorescence showing early hyperautofluorescence at the injection site, heterogeneous autofluorescence in the detached area and hypoautofluorescence annulus at the inner edge of the bleb. **C.** Near-infrared autofluorescence showing early hyperautofluorescence at the foveal location (D3), progressively returning to a normal inner aspect with persisting demarcation line (M4). Scale bars: 200µm.
