# Supplementary material for: Adaptive Optics Flood Illumination Ophthalmoscopy in Nonhuman Primates: Findings in Normal and Short-term Induced Detached Retinae
Source: Ophthalmol Sci. 2023 Apr 20;3(4):100316. doi: 10.1016/j.xops.2023.100316 (PMC10238594; doi:10.1016/j.xops.2023.100316)
Supplement: Figure S3 — Sample of adaptive optics flood illumination ophthalmoscopy (AO-FIO) in nonhuman primate 2 (NHP2) showing the retinal vasculature landmarks allowing to follow-up changes after retinal detachment (RD) at the same location. A, Infrared imaging of the left eye before induced RD in NHP2. AO-FIO imaging samples are overlaid to show that the vasculature of the primate is well recognized and can ensure repeatability. B–D, Sample of AO-FIO imaging at 2° of superior eccentricity at baseline (B), 2 months (C), and 4 months (D) after short-term induced RD. The same recognizable vessel is spotted (white arrow). The superior foveal area is shown (white star). Scale bars: A: 200 μm, B–D: 120 μm. [file mmc3.pdf]

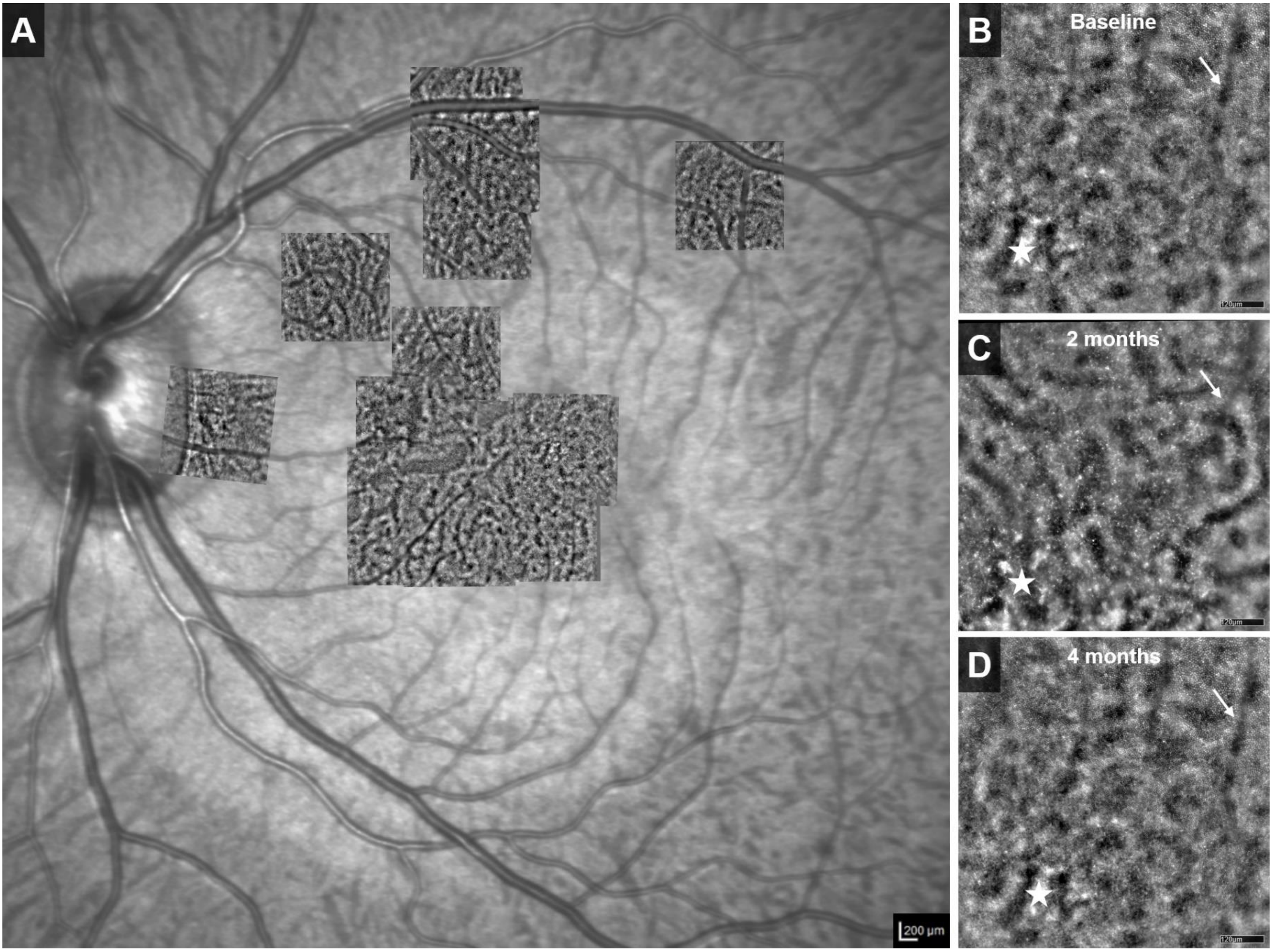

Figure S3. Sample of adaptive optics flood illumination ophthalmoscopy (AO-FIO) in non-human primate 2 (NHP2) showing the retinal vasculature landmarks allowing to follow up changes after retinal detachment (RD) at the same location. A. Infrared imaging of the left eye prior to induced RD in NHP2. AO-FIO imaging samples are overlaid to show that the vasculature of the primate is well recognized and can ensure repeatability. **B-D.** Sample of AO-FIO imaging at 2° of superior eccentricity at baseline (**B**), 2 months (**C**) and 4 months (**D**) after short-term induced RD. The same recognizable vessel is spotted (white arrow). The superior foveal area is shown (white star).

Scale bars: A: 200µm, B-D: 120µm.
