# Supplementary material for: Adaptive Optics Flood Illumination Ophthalmoscopy in Nonhuman Primates: Findings in Normal and Short-term Induced Detached Retinae
Source: Ophthalmol Sci. 2023 Apr 20;3(4):100316. doi: 10.1016/j.xops.2023.100316 (PMC10238594; doi:10.1016/j.xops.2023.100316)
Supplement: Figure S4 — Sample of adaptive optics flood illumination imaging (AO-FIO) of the photoreceptor layer in nonhuman primate 1 (NHP1) and human 1 according to horizontal eccentricity (away from the optic disk) with corresponding Yellott’s ring and power spectrum. Note that the Yellott’s ring gets smaller and thinner with eccentricity in both the NHP and human. Similarly, the cone mosaic peak (black arrow) on the power spectrum sets at decreasing spatial frequencies with eccentricity. Interestingly, the peak looks sharper in the NHP in all eccentricities, suggesting that cone reflectivity is more homogeneous in NHP than in human. Despite that the power spectrum plots are almost totally overlapping in the 2 species, a likely artifactual shift between spatial frequencies at peak can be observed between 2- and 4-degrees. Scale bars: NHP: 30 μm, human: 40 μm. [file mmc4.pdf]

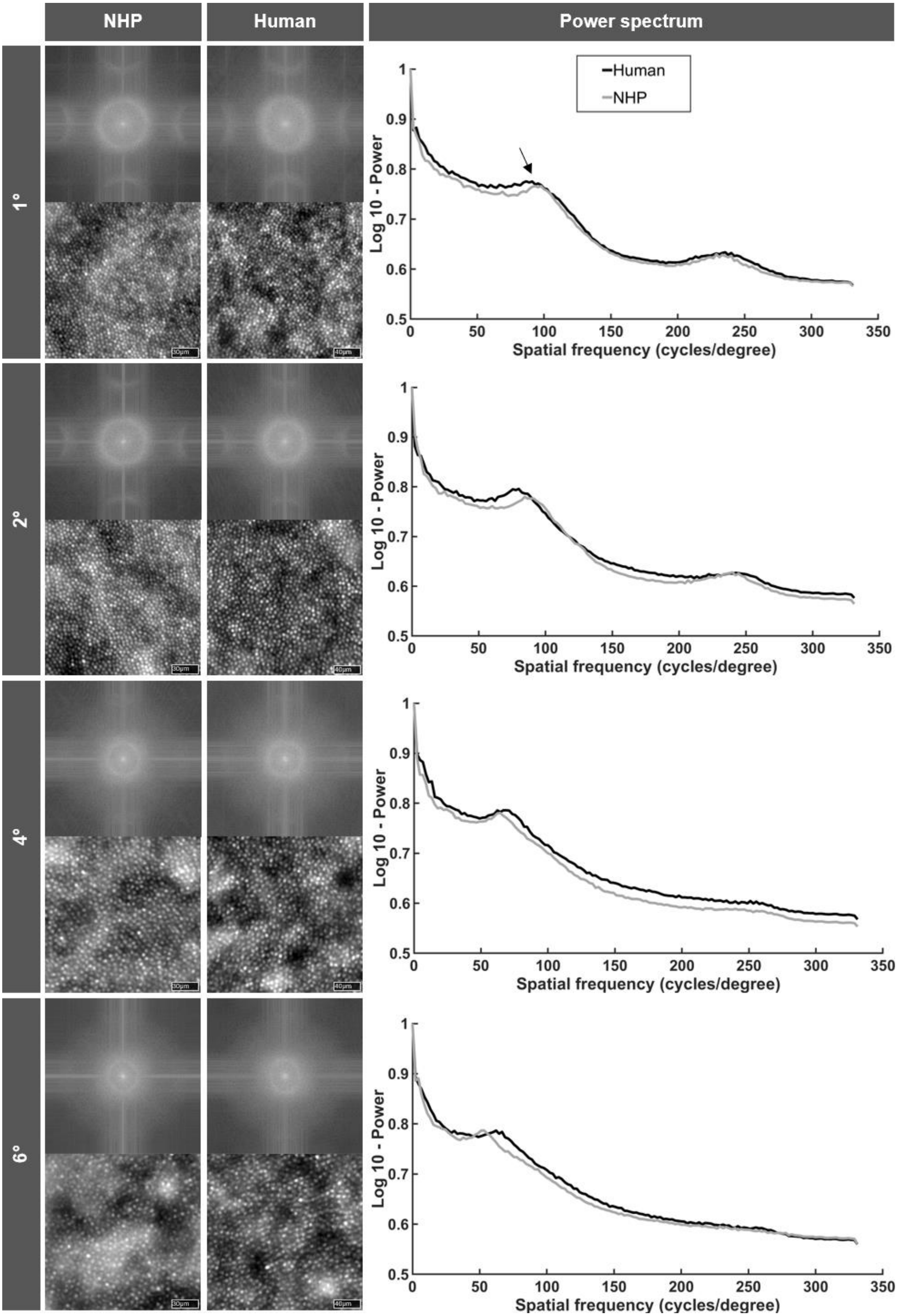
NHP
Human
Power spectrum
1°
2°
4°
6°
Human
NHP
Log 10 - Power
Spatial frequency (cycles/degree)
30µm
40µm

Figure S4. Sample of adaptive optics flood illumination imaging (AO-FIO) of the photoreceptor layer in non-human primate 1 (NHP1) and human 1 according to horizontal eccentricity (away from the optic disk) with corresponding Yellott's ring and power spectrum. Note that the Yellott's ring gets smaller and thinner with eccentricity in both NHP and human. Similarly, the cone mosaic peak (black arrow) on the power spectrum sets at decreasing spatial frequencies with eccentricity. Interestingly, the peak looks sharper in NHP in all eccentricities, suggesting that cone reflectivity is more homogeneous in NHP than in human. Despite that the power spectrum plots are almost totally overlapping in the two species, a likely artifactual shift between spatial frequencies at peak can be observed between 2- and 4-degree.

Scale bars: NHP: 30µm, human: 40µm.
