# Supplementary material for: Adaptive Optics Flood Illumination Ophthalmoscopy in Nonhuman Primates: Findings in Normal and Short-term Induced Detached Retinae
Source: Ophthalmol Sci. 2023 Apr 20;3(4):100316. doi: 10.1016/j.xops.2023.100316 (PMC10238594; doi:10.1016/j.xops.2023.100316)
Supplement: Figure S5 — Sample of power spectrum changes after short-term retinal detachment (RD) in nonhuman primate 1 (NHP1). A, Merged power spectrum plots of follow-up retinal adaptive optics flood illumination (AO-FIO) 300 × 300 pixel images at 2-degrees of temporal eccentricity in NHP1. Note the progressive normalization of the power spectrum. The black dashed line indicates the peak spatial frequency at baseline, also reached at M4. B, Sample 4 × 4-degree AO-FIO imaging of the photoreceptor layer at the edge of the detached area 15 days after the reattachment and the corresponding power spectrum in 300 × 300 pixels windows (gray window and plot: detached area, black window and plot: nondetached area). The dashed white line highlights the borderline between the detached and nondetached areas. The dark line on the power spectrum plot indicates the peak spatial frequency at the nondetached area window. Scale bar: 120 μm. [file mmc5.pdf]

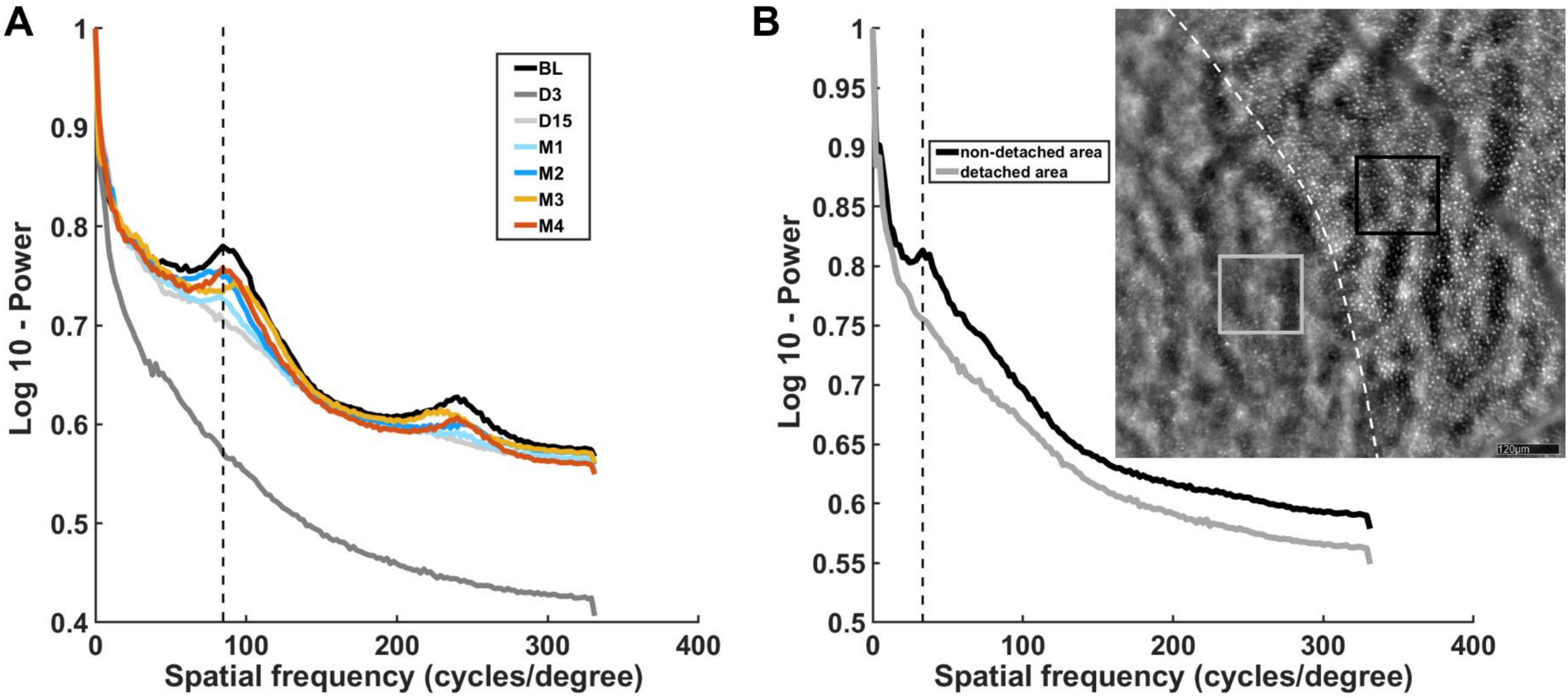

Figure S5. Sample of power spectrum changes following short-term retinal detachment (RD) in Non-human primate 1 (NHP1). A. Merged power spectrum plots of follow-up retinal adaptive optics flood illumination (AO-FIO) 300 x 300 pixels images at 2-degree of temporal eccentricity in NHP1. Note the progressive normalization of the power spectrum. The black dashed line indicates the peak spatial frequency at baseline, also reached at M4. **B.** Sample 4 x 4-degree AO-FIO imaging of the photoreceptor layer at the edge of the detached area 15 days after the reattachment and the corresponding power spectrum in 300 x 300 pixels windows (grey window and plot: detached area, black window and plot: non-detached area). The dashed white line highlights the borderline between the detached and non-detached areas. The dark line on the power spectrum plot indicates the peak spatial frequency at the non-detached area window.

Scale bar: 120µm.
