# Supplementary material for: Adaptive Optics Flood Illumination Ophthalmoscopy in Nonhuman Primates: Findings in Normal and Short-term Induced Detached Retinae
Source: Ophthalmol Sci. 2023 Apr 20;3(4):100316. doi: 10.1016/j.xops.2023.100316 (PMC10238594; doi:10.1016/j.xops.2023.100316)
Supplement: Figure S7 — Additional adaptive optics flood illumination (AO-FIO) photoreceptor layer imaging and features in nonhuman primates (NHPs). A, AO-FIO photoreceptor layer imaging in the right eye of NHP1 at the upper temporal branches division (8 x 4-degree field, montage using i2K retina AO, DualAlign LCC, Troy, NY). B, Corresponding AO-FIO field showing the ‘edge effects’ of different sized vessels: totally masked cones at the edge of major vessels (arrows) and partially masked cones at the edge of thinner vessels (arrowhead). C, AO-FIO imaging of the temporal side of the optic disk rim (left eye), a perioptic 50-60 μm rim free of distinguishable cones. Scale bars: A–C: 120 μm, highlighted area in C: 30 μm. [file mmc6.pdf]

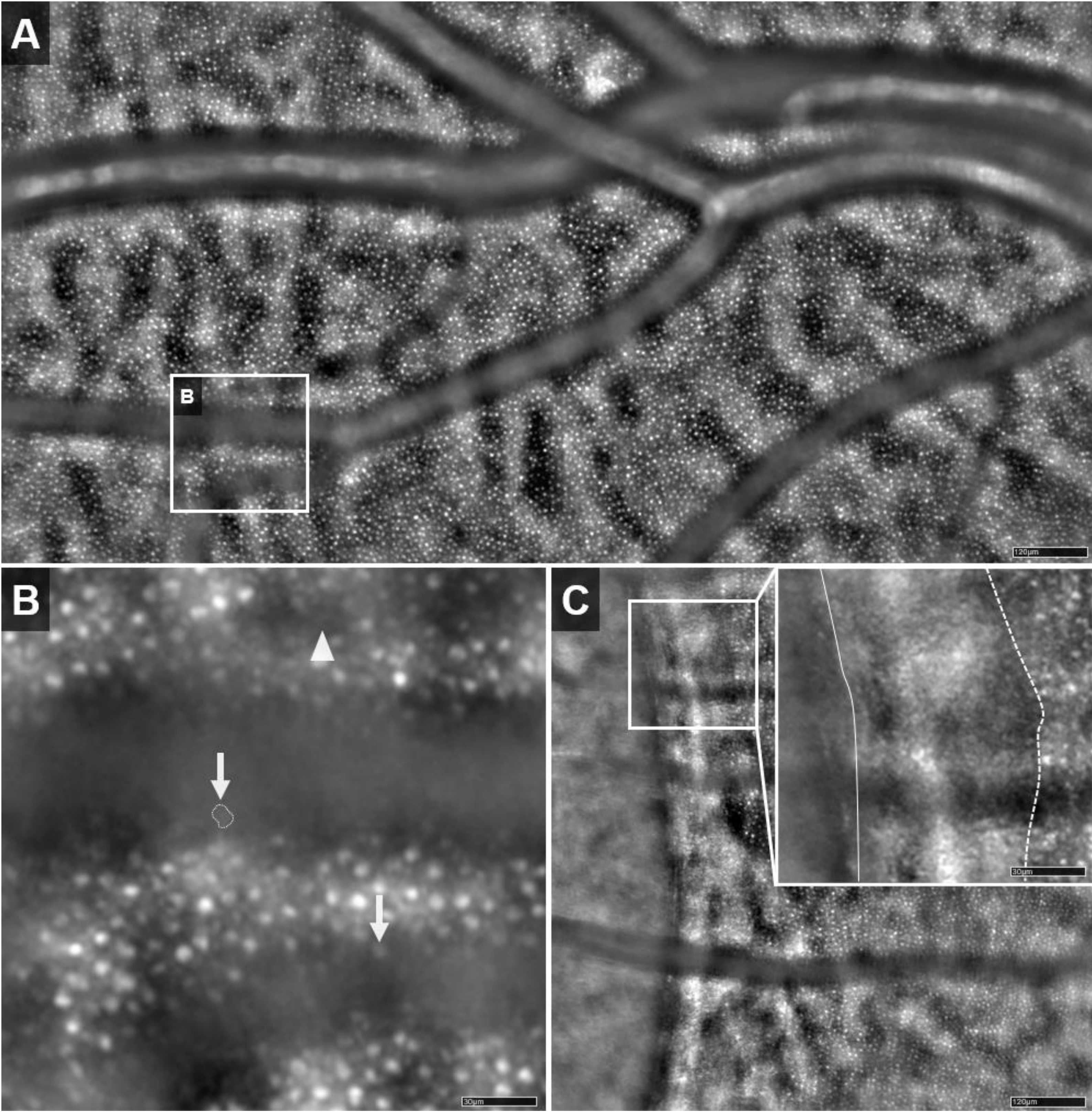

Figure S7. Additional adaptive optics flood illumination (AO-FIO) photoreceptor layer imaging and features in non-human primates (NHP). A. AO-FIO photoreceptor layer imaging in the right eye of NHP1 at the upper temporal branches division (8 x 4-degree field, montage using i2K retina AO, DualAlign LCC, Troy, NY). **B.** Corresponding AO-FIO field showing the 'edge effects' of different sized vessels: totally masked cones at the edge of major vessels (arrows) and partially masked cones at the edge of thinner vessels (arrowhead). **C.** AO-FIO imaging of the temporal side of the optic disk rim (left eye) a perioptic 50-60µm rim free of distinguishable cones.

Scale bars: A-C: 120µm, highlighted area in C: 30µm.
