# Supplementary material for: Adaptive Optics Flood Illumination Ophthalmoscopy in Nonhuman Primates: Findings in Normal and Short-term Induced Detached Retinae
Source: Ophthalmol Sci. 2023 Apr 20;3(4):100316. doi: 10.1016/j.xops.2023.100316 (PMC10238594; doi:10.1016/j.xops.2023.100316)
Supplement: Tables S3–S6 [file mmc7.pdf]

| Values | Horizontal eccentricity (degrees) | NHP1<br>5 Y/O male | NHP2<br>5 Y/O male | NHP3<br>5 Y/O male | NHP4<br>15 Y/O female | Mean NHP values<br>± SD |
|---|---|---|---|---|---|---|
| **Cone density (cones/deg$^2$)** | **1°** | 2436 | 2391 | 2402 | 2424 | **2413** ± 20 |
| | **2°** | 2113 | 2228 | 2105 | 2225 | **2168** ± 68 |
| | **4°** | 1592 | 1554 | 1602 | 1594 | **1586** ± 21 |
| | **6°** | 1333 | 1318 | 1301 | 1344 | **1324** ± 51 |
| **Cone mosaic regularity (%)** | **1°** | 97.77 | 96.26 | 94.80 | 97.13 | **96.49** ± 1.29 |
| | **2°** | 100.00 | 96.92 | 98.25 | 98.96 | **98.53** ± 1.29 |
| | **4°** | 98.88 | 99.90 | 98.99 | 100 | **99.44** ± 0.59 |
| | **6°** | 96.54 | 95.68 | 96.28 | 97.31 | **96.45** ± 0.68 |
| **Cone spacing (arcmin)** | **1°** | 1.33 | 1.36 | 1.34 | 1.29 | **1.33** ± 0.03 |
| | **2°** | 1.41 | 1.45 | 1.44 | 1.40 | **1.43** ± 0.02 |
| | **4°** | 1.60 | 1.59 | 1.63 | 1.61 | **1.61** ± 0.02 |
| | **6°** | 1.84 | 1.86 | 1.90 | 1.81 | **1.85** ± 0.04 |

**Table 3. Individual cone mosaic metrics values in non-human primates (NHP) along the horizontal axis (toward the temporal retina).** Mean values are shown in bold. SD: standard deviation.

| Values | Horizontal eccentricity (degrees) | Human 1 27 Y/O male | Human 2 38 Y/O male | Human 3 58 Y/O male | Human 4 25 Y/O female | Mean human values ± SD |
|---|---|---|---|---|---|---|
| **Cone density (cones/deg$^2$)** | **1°** | 2401 | 2424 | 2494 | 2503 | **2456** ± 51 |
| | **2°** | 2180 | 2257 | 2145 | 2226 | **2202** ± 49 |
| | **4°** | 1854 | 1802 | 1694 | 1767 | **1779** ± 67 |
| | **6°** | 1275 | 1305 | 1413 | 1389 | **1346** ± 66 |
| **Cone mosaic regularity (%)** | **1°** | 91.13 | 92.45 | 92.80 | 91.26 | **91.91** ± 0.84 |
| | **2°** | 98.02 | 98.71 | 97.92 | 96.48 | **97.78** ± 0.94 |
| | **4°** | 99.95 | 100.00 | 99.99 | 100.00 | **99.99** ± 0.02 |
| | **6°** | 95.68 | 94.69 | 95.45 | 95.31 | **95.28** ± 0.42 |
| **Cone spacing (arcmin)** | **1°** | 1.37 | 1.35 | 1.34 | 1.32 | **1.35** ± 0.02 |
| | **2°** | 1.40 | 1.41 | 1.43 | 1.41 | **1.41** ± 0.01 |
| | **4°** | 1.48 | 1.50 | 1.54 | 1.52 | **1.51** ± 0.03 |
| | **6°** | 1.85 | 1.82 | 1.77 | 1.79 | **1.81** ± 0.04 |

**Table 4. Individual cone mosaic metrics values in humans along the horizontal axis (toward the temporal retina).** Mean values are shown in bold. SD: standard deviation.

| Values | Vertical eccentricity (degrees) | NHP1<br>5 Y/O male | NHP2<br>5 Y/O male | NHP3<br>5 Y/O male | NHP4<br>15 Y/O female | Mean NHP values<br>± SD |
|---|---|---|---|---|---|---|
| **Cone density ($cones/deg^2$)** | **1°** | 2352 | 2318 | 2322 | 2299 | **2323** ± 22 |
| | **2°** | 2084 | 2126 | 2129 | 2214 | **2138** ± 55 |
| | **4°** | 1496 | 1517 | 1504 | 1493 | **1503** ± 11 |
| | **6°** | 1118 | 1201 | 1197 | 1222 | **1185** ± 46 |
| **Cone mosaic regularity (%)** | **1°** | 96.30 | 97.22 | 96.18 | 97.68 | **96.85** ± 0.73 |
| | **2°** | 98.19 | 99.54 | 98.37 | 97.14 | **98.31** ± 0.98 |
| | **4°** | 99.25 | 99.47 | 98.9 | 100 | **99.41** ± 0.46 |
| | **6°** | 95.86 | 96.28 | 97.14 | 96.95 | **92.49** ± 0.75 |
| **Cone spacing (arcmin)** | **1°** | 1.36 | 1.37 | 1.36 | 1.32 | **1.35** ± 0.02 |
| | **2°** | 1.47 | 1.48 | 1.44 | 1.44 | **1.46** ± 0.02 |
| | **4°** | 1.59 | 1.63 | 1.61 | 1.6 | **1.61** ± 0.02 |
| | **6°** | 1.93 | 1.94 | 1.92 | 1.92 | **1.93** ± 0.01 |

**Table 5. Individual cone mosaic metrics values in non-human primates (NHP) along the vertical axis (toward the superior retina).** Mean values are shown in bold. SD: standard deviation.

| Values | Vertical eccentricity (degrees) | Human 1 27 Y/O male | Human 2 38 Y/O male | Human 3 58 Y/O male | Human 4 25 Y/O female | Mean human values ± SD |
|---|---|---|---|---|---|---|
| **Cone density (cones/deg$^2$)** | **1°** | 2395 | 2401 | 2420 | 2467 | **2421** ± 33 |
| | **2°** | 2124 | 2142 | 2098 | 2183 | **2137** ± 36 |
| | **4°** | 1526 | 1489 | 1478 | 1503 | **1499** ± 21 |
| | **6°** | 1257 | 1265 | 1216 | 1303 | **1260** ± 36 |
| **Cone mosaic regularity (%)** | **1°** | 92.91 | 92.85 | 92.84 | 91.36 | **92.49** ± 0.75 |
| | **2°** | 97.26 | 98.61 | 97.36 | 98.52 | **97.94** ± 0.73 |
| | **4°** | 100 | 99.67 | 100 | 100 | **99.92** ± 0.17 |
| | **6°** | 96.55 | 93.99 | 95.58 | 95.49 | **95.40** ± 1.06 |
| **Cone spacing (arcmin)** | **1°** | 1.37 | 1.37 | 1.35 | 1.37 | **1.37** ± 0.01 |
| | **2°** | 1.42 | 1.43 | 1.39 | 1.43 | **1.42** ± 0.02 |
| | **4°** | 1.55 | 1.58 | 1.54 | 1.55 | **1.56** ± 0.02 |
| | **6°** | 1.90 | 1.89 | 1.79 | 1.83 | **1.85** ± 0.05 |

**Table 6. Individual cone mosaic metrics values in humans along the vertical axis (toward the superior retina).** Mean values are shown in bold. SD: standard deviation.
